# Supplementary material for: Dual lineage origins contribute to neocortical astrocyte diversity
Source: Nat Commun. 2025 Jul 30;16:6992. doi: 10.1038/s41467-025-61829-4 (PMC12310952; doi:10.1038/s41467-025-61829-4)
Supplement: Supplementary file 1 — Supplementary Information [file 41467_2025_61829_MOESM1_ESM.pdf]

Supplementary information

Dual lineage origins contribute to neocortical astrocyte diversity

Jiafeng Zhou[1], Ilaria Vitali[2], Sergi Roig-Puiggros[1], Awais Javed[1], Iva Cantando[3], Matteo Puglisi[4,5], Paola Bezzi[3,6], Denis Jabaudon[1], Christian Mayer[2], Riccardo Bocchi[1,*]

[1]Department of Basic Neurosciences, University of Geneva, Geneva, Switzerland
[2]Max Planck Institute for Biological Intelligence, Martinsried, Germany
[3]Department of Fundamental Neurosciences, University of Lausanne, Lausanne, Switzerland
[4]Division of Physiological Genomics, Biomedical Center, Ludwig-Maximilians-Universität München, Planegg-Martinsried, Germany.
[5]Institute for Stem Cell Research, Helmholtz Zentrum München Deutsches Forschungszentrum für Gesundheit und Umwelt (GmbH), Nuremberg, Germany.
[6]Department of Physiology and Pharmacology, Sapienza University of Rome, Rome, Italy

*Correspondence: riccardo.bocchi@unige.ch

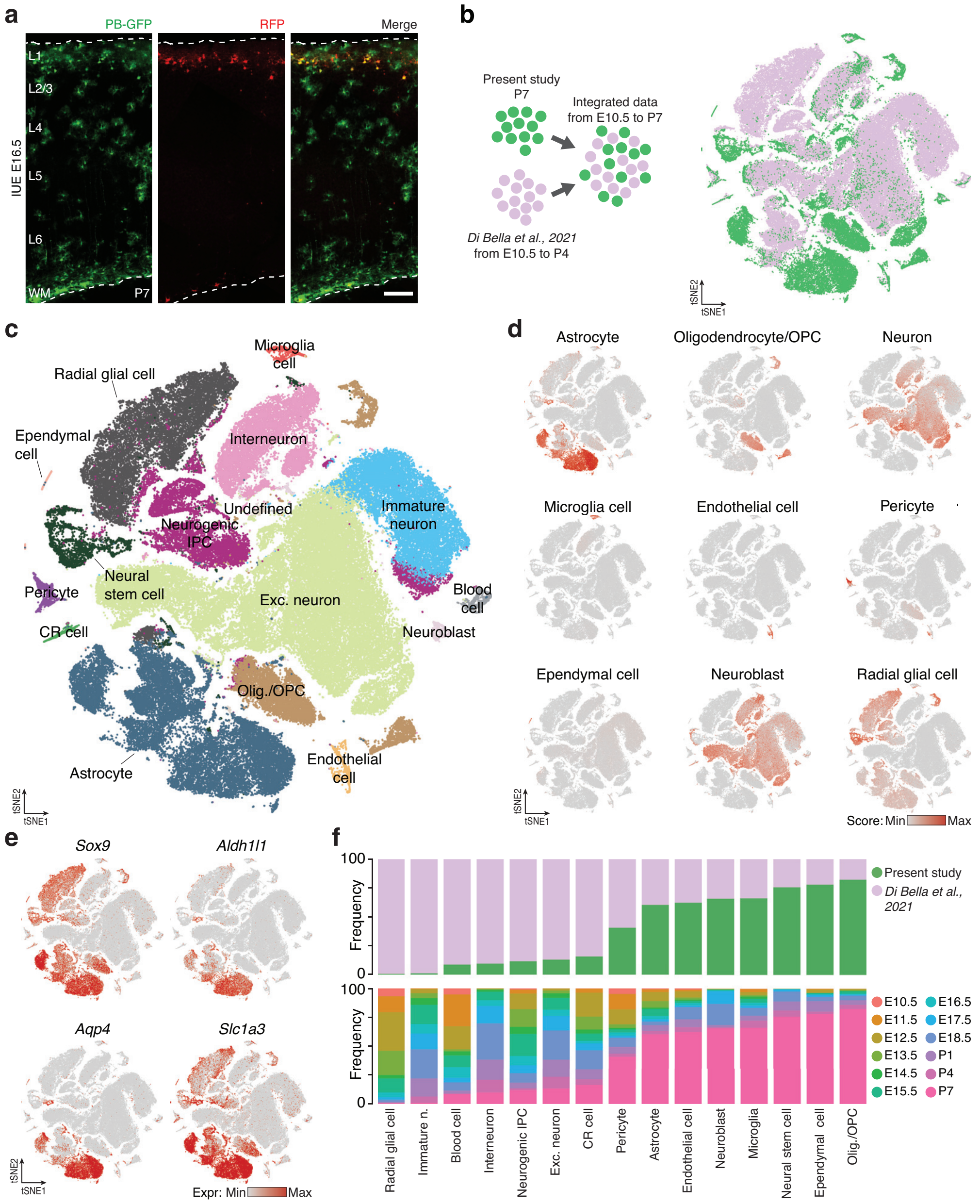

Supplementary Fig. 1 | Integration and annotation of mouse embryonic and postnatal scRNA-seq datasets. a, P7 cortical column electroporated at E16.5 with integrative (PB-GFP) and episomal (RFP) plasmids. Scale bar: 100 µm. **b-c,** tSNE plots of the integrated dataset showing cells from the present study (green) and *Di Bella et al.* (pink) (b), and corresponding cell type identities (c). **d**, tSNE plots showing cell type scores, calculated based on known markers for major cell types in the neocortex (full gene list in Supplementary Table 1). **e**, tSNE plots showing the expression of four canonical astrocytic markers in the integrated dataset. **f**, Proportion of cells from the present study and *Di Bella et al*. (top), or from different developmental stages (bottom), for each cell type. CR cell: Cajal–Retzius cell; IPC: intermediate progenitor cell; Olig./OPC: oligodendrocyte and oligodendrocyte precursor cell; Exc. Neuron: excitatory neuron; IUE: *in utero* electroporation; WM: white matter; L: layer.

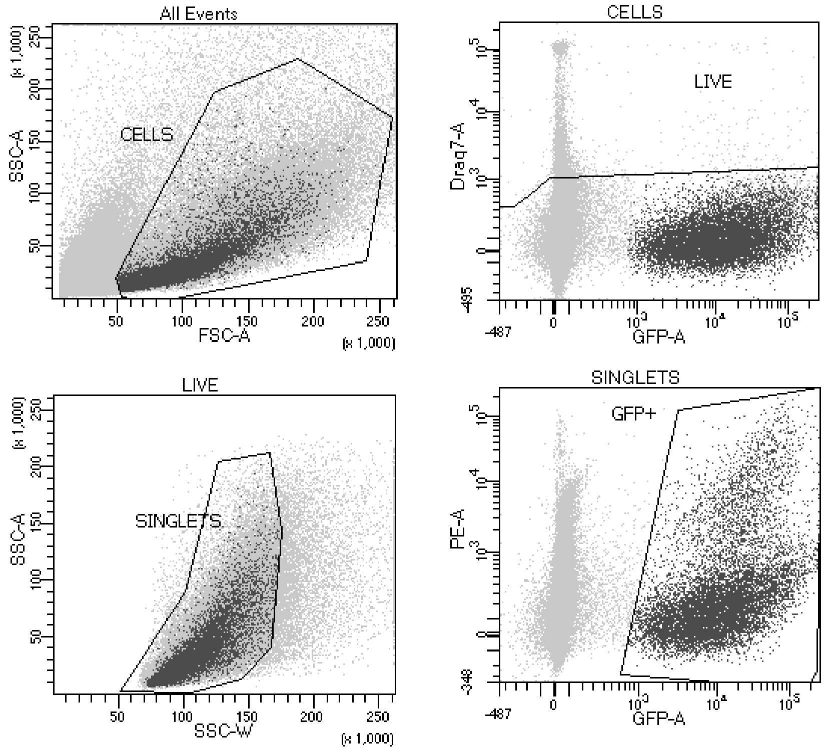

Supplementary Fig. 2 | Representative FACS gating strategy for GFP⁺ electroporated cells. Flow cytometry plots illustrating the gating strategy used to isolate GFP⁺ electroporated cells dissociated from dissected mouse cortices. This approach was consistently applied across all scRNA-seq experiments performed in the study.

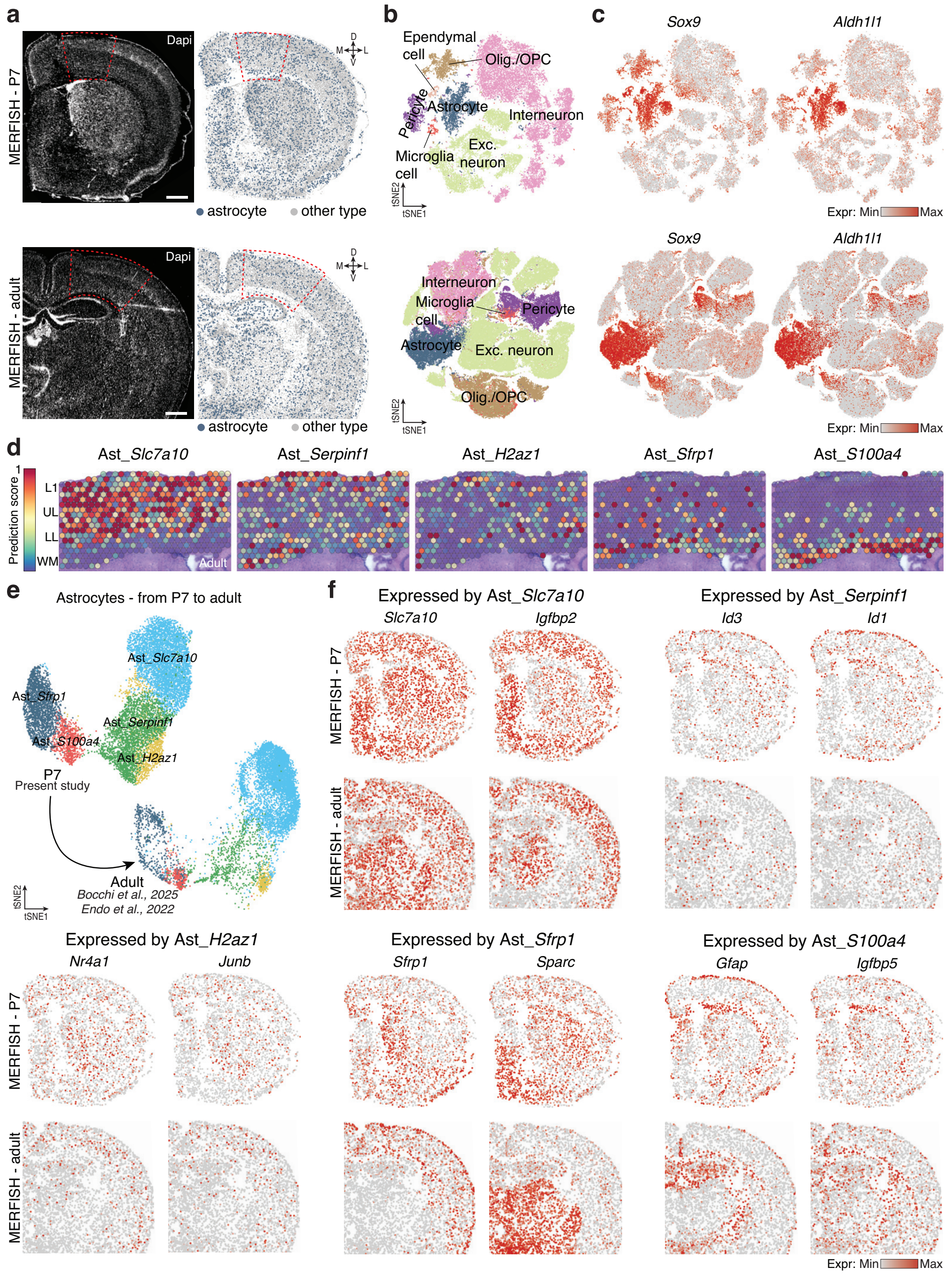

Supplementary Fig. 3 | Mouse P7 and adult cortical spatial transcriptomic datasets. a, Left: coronal section used to generate the P7 (top) and adult (bottom) MERFISH datasets. The dashed region corresponds to the analyzed area in Fig. 1e and 1f. Right: spatial positioning of each detected cell, astrocytes are highlighted in blue. Scale bars: 200 µm. **b**, tSNE plot showing all cell types found in the P7 (top) and adult (bottom) MERFISH datasets. **c**, tSNE plots of the P7 (top) and adult (bottom) MERFISH dataset showing the expression of two canonical astrocytes markers. **d**, Spatial distribution of the five astrocyte clusters using a publicly available Visium dataset of a mouse sagittal section, illustrating their predicted localization within the cortical column. **e**, Label transfer of the five astrocyte subtypes from the P7 scRNA-seq dataset generated in this study to an adult scRNA-seq dataset, obtained by merging two distinct publicly available datasets. **f**, Spatial expression of representative genes for each astrocyte subtype. Genes are derived from the differential gene expression analysis presented in Fig. 1c (full gene list in Supplementary Data 1). L1: layer 1; UL: upper layer; LL: lower layer; WM: white matter.

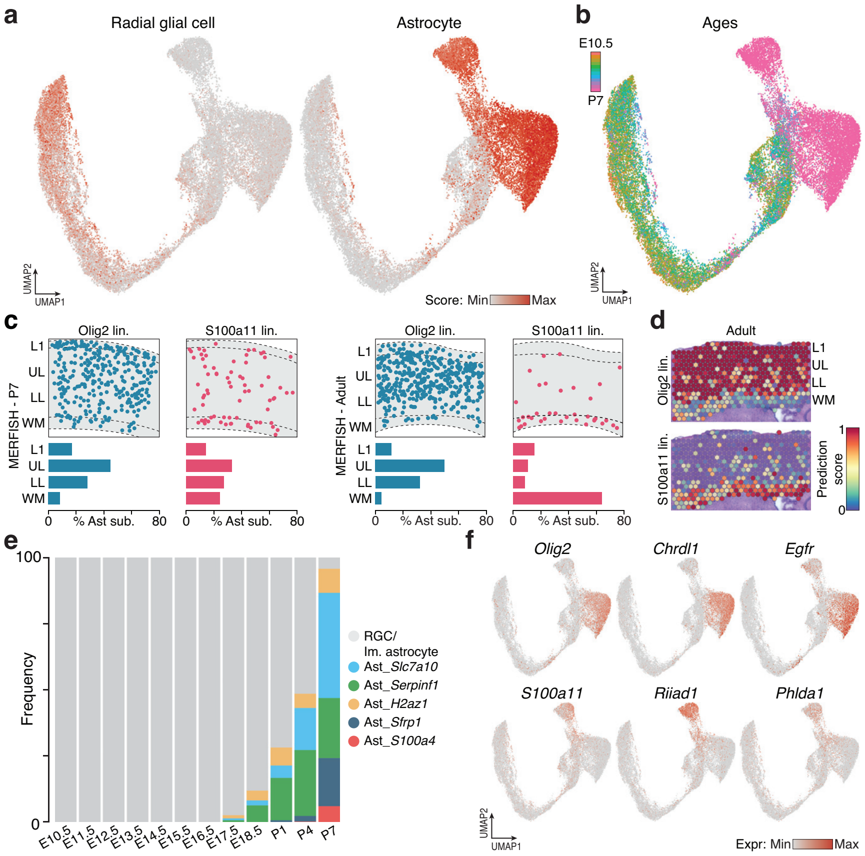

Supplementary Fig. 4 | Characterization of cortical astrocyte trajectories. a, UMAP representation of radial glial cell and astrocyte scores. **b**, UMAP representation of RGCs and astrocytes from E10.5 to P7 color-coded according to their ages. **c**, Spatial positioning of astrocyte lineages (top) and their distribution along the cortical column (bottom) in P7 (left, cropped from Supplementary Fig. 2a top) and adult (right, cropped from Supplementary Fig. 2a bottom) MERFISH coronal sections. **d**, Spatial distribution of the astrocyte lineages using a publicly available Visium dataset of a mouse sagittal section. **e**, Relative proportion of the different astrocyte subtypes over time. **f**, UMAP plots showing the expression of selected trajectory-specific genes. L1: layer 1; UL: upper layer; LL: lower layer; WM: white matter.

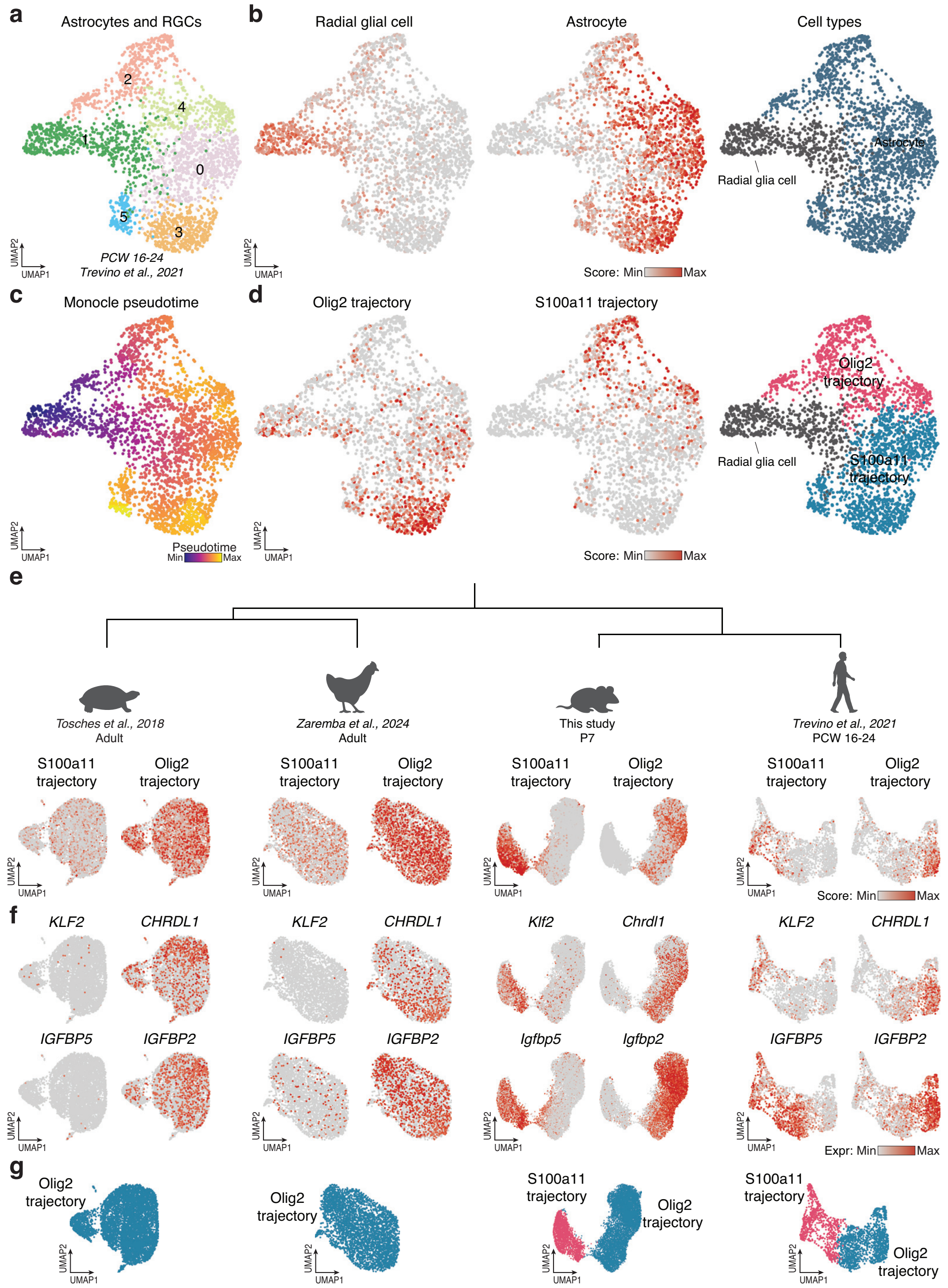

Supplementary Fig. 5 | Astrocytes from the S100a11 lineage represent an evolutionary acquisition in the development of the mammalian neocortex. a, UMAP plot illustrating the cluster analysis of RGCs and astrocytes in developing human neocortex. **b**, UMAP representation of radial glial cell and astrocyte scores (left) and annotated cell types (right) in the human dataset (full gene list in Supplementary Table 1). **c**, UMAP plot showing Monocle pseudotime, depicting the developmental trajectory from RGCs to astrocyte subtypes in the human neocortex. **d**, UMAP representation of S100a11 and Olig2 trajectory scores (left) and annotated astrocyte trajectories (right), based on trajectory-specific genes (full gene list in Supplementary Data 2) in the human dataset. **e-g**, UMAP plots of S100a11 and Olig2 trajectory scores (**e**), expression of four representative genes (**f**, specific to S100a11 and Olig2 trajectory), and astrocyte trajectories (**g**), comparing four species (turtle, bird, mouse, and human).

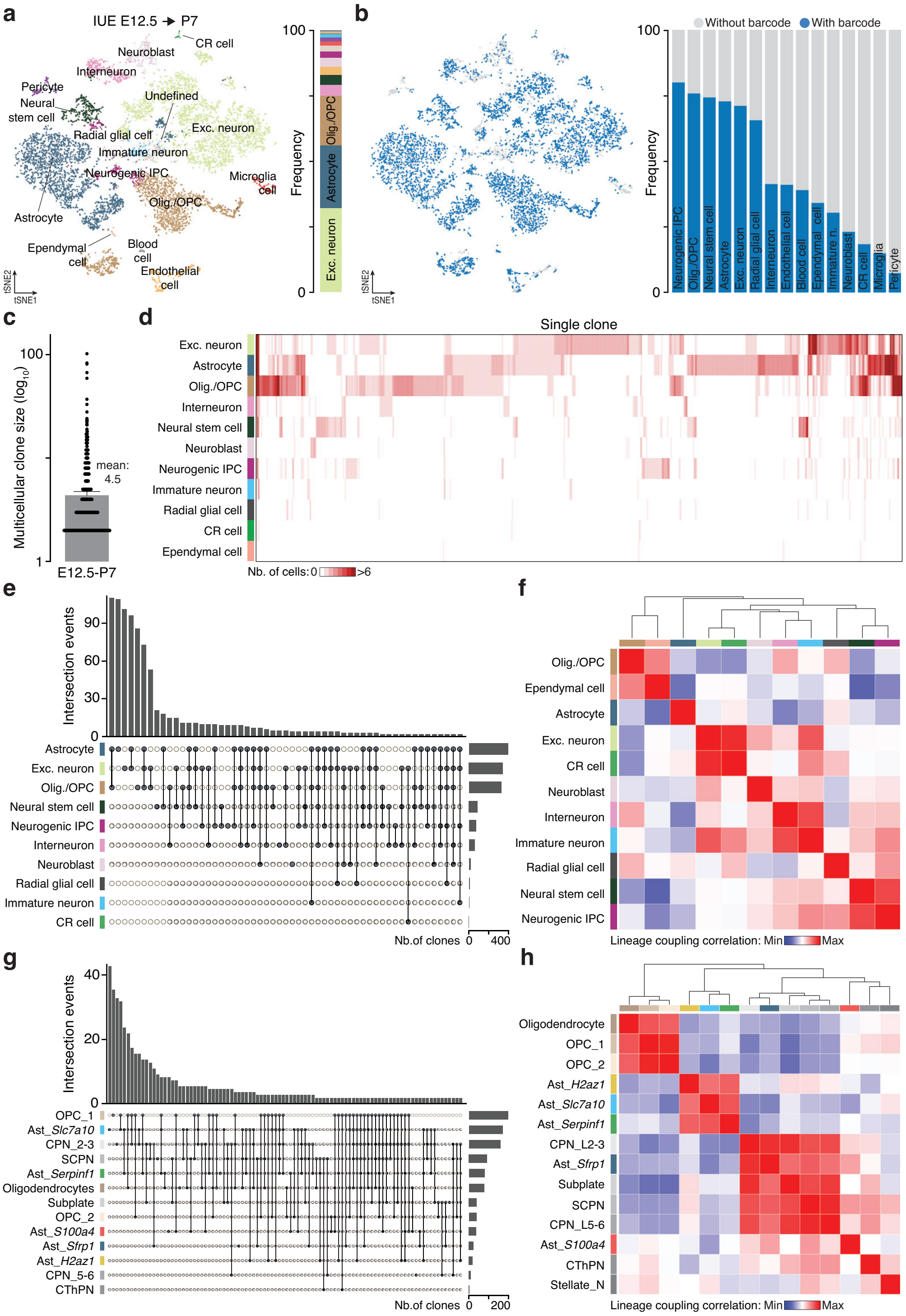

Supplementary Fig. 6 | Lineage coupling analyses of major cell types in the mouse neocortex. a, Left: tSNE representation of all major cell types traced from E12.5 and collected at P7. Right: proportion of each cell type. **b**, tSNE plot highlighting cells tagged by TrackerSeq barcodes (left) with the relative proportion of cells with and without barcodes in each major cell type (right). **c**, Quantification of the average clone size. Values are shown as mean ± s.e.m.; n = 958 clones. **d**, Clonal distributions across major cell types. Each column represents one clone, with number of cells per clone indicated by color scale. Blood cell, Microglia cell, Pericyte and Endothelial cell are excluded from the distribution analyses. **e**, UpSet plot showing the number of clones shared or unique between all major cell types. Top bar graphs indicate observed intersections; right bar graphs show clone counts per major cell type. **f**, Heatmap of lineage coupling scores between pairs of cell types included in (d). Values range from positive (red, coupled) to negative (blue, anti-coupled). **g**, UpSet plot showing the number of clones shared or unique between astrocyte, oligodendrocytes/OPCs and excitatory neuron subtypes. Top bar graphs indicate observed intersections; right bar graphs show clone counts per cell subtype. **h**, Heatmap of lineage coupling scores between pairs of cell types further subdivided in subtypes (*e.g.,* astrocyte subtypes, excitatory neuron subtypes and oligodendrocytes/OPCs subtypes). Values range from positive (red, coupled) to negative (blue, anti-coupled). IUE: *in utero* electroporation.

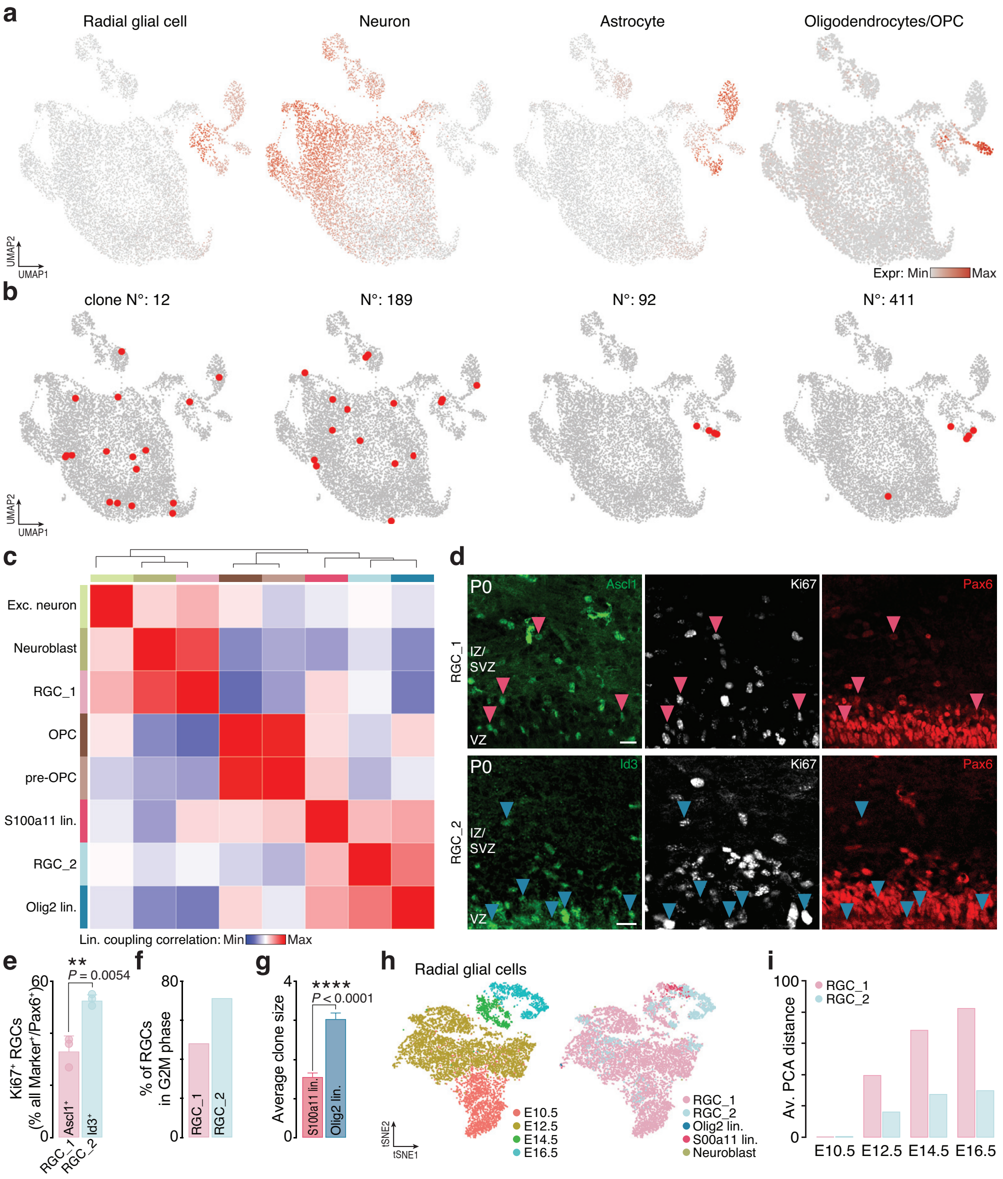

Supplementary Fig. 7 | Identification and characterization of cortical RGC subtypes in scRNA-seq datasets. a, UMAP representation of radial glial cell, neuron, astrocyte and oligodendrocytes/OPCs scores in the E18.5 dataset. **b**, Examples of clones that are either shared between neurons, RGC_1 and S100a11 lineage astrocytes (left) or restricted within Olig2 lineage astrocytes and RGC_2 (right). **c**, Heatmap of lineage coupling scores between pairs of major cell types identified in the E18.5 dataset. Values range from positive (red, coupled) to negative (blue, anti-coupled). **d**, Ascl1 (top) and Id3 (bottom) staining in Pax6$^+$ RGCs within the VZ/SVZ, co-stained with the cell cycle marker Ki67. Scale bars: 50 µm. **e,** Fraction of Ascl1$^+$/Pax6$^+$ and Id3$^+$/Pax6$^+$ RGCs in the cell cycle (Ki67$^+$). n = 3 animals; two-tailed t-test. **f**, Fraction of RGCs in the G2/M phase (see Materials and Methods section for details on the analysis). **g**, Average clone size of the S100a11 and Olig2 lineages. n = 68 clones (S100a11 lin.) and 275 clones (Olig2 lin.); two-tailed t-test. **h**, tSNE plots showing radial glial cells from *Di Bella et al.* at E10.5, E12.5, E14.5 and E16.5, color-coded according to their ages (left) and by cell types predicted from the E18.5 dataset (right). **i**, Average PCA distance within each RGC subtypes. The E10.5 time point was used as reference for each RGC subtypes and the distance E10.5 to E12.5, E10.5 to E14.5 and E10.5 to E16.5 are represented. **p<0.01, ****p<0.0001. Values are shown as mean ± s.d.. VZ: ventricular zone; SVZ: subventricular zone; IZ: intermediate zone. Source data are provided as a Source Data file.

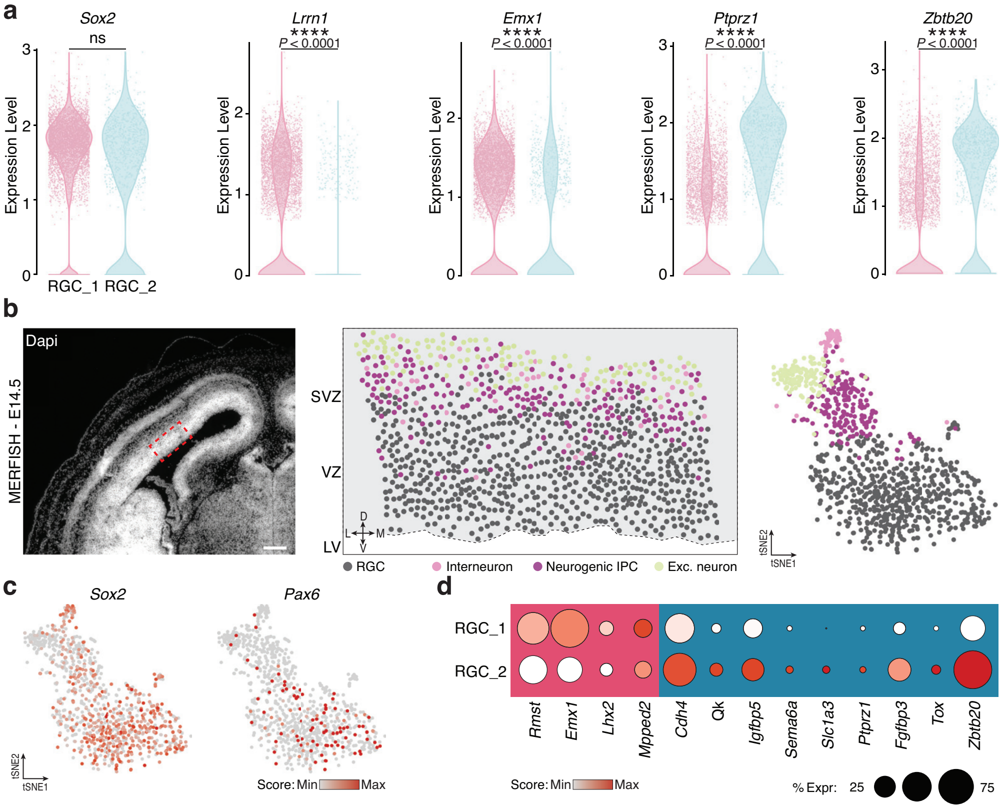

Supplementary Fig. 8 | Molecular and spatial distribution of cortical RGC subtypes. a, Expression of selected genes distinguishing RGC_1 and RGC_2 at E12.5, based on Fig. 3j. No significant difference in Sox2 expression was observed, confirming the identity of RGC_1 and RGC_2 as radial glial cell. Two-sided Mann–Whitney U-test. **b**, Left: coronal section used to generate the E14.5 MERFISH dataset. The dashed region corresponds to the analyzed area in the middle. Middle: spatial positioning of each detected cell. Right: tSNE plot showing all cell types found in the E14.5 MERFISH dataset. Scale bar: 200 µm. **c**, tSNE plots showing the expression of two RGC markers, *Sox2* and *Pax6*, in the E14.5 MERFISH dataset. **d**, Expression level of selected genes in MERFISH dataset (full gene list in Supplementary Data 3) for each RGC subtypes. n.s. not significant, ****$p< 0.0001$. VZ: ventricular zone; SVZ: subventricular zone; LV: lateral ventricle.

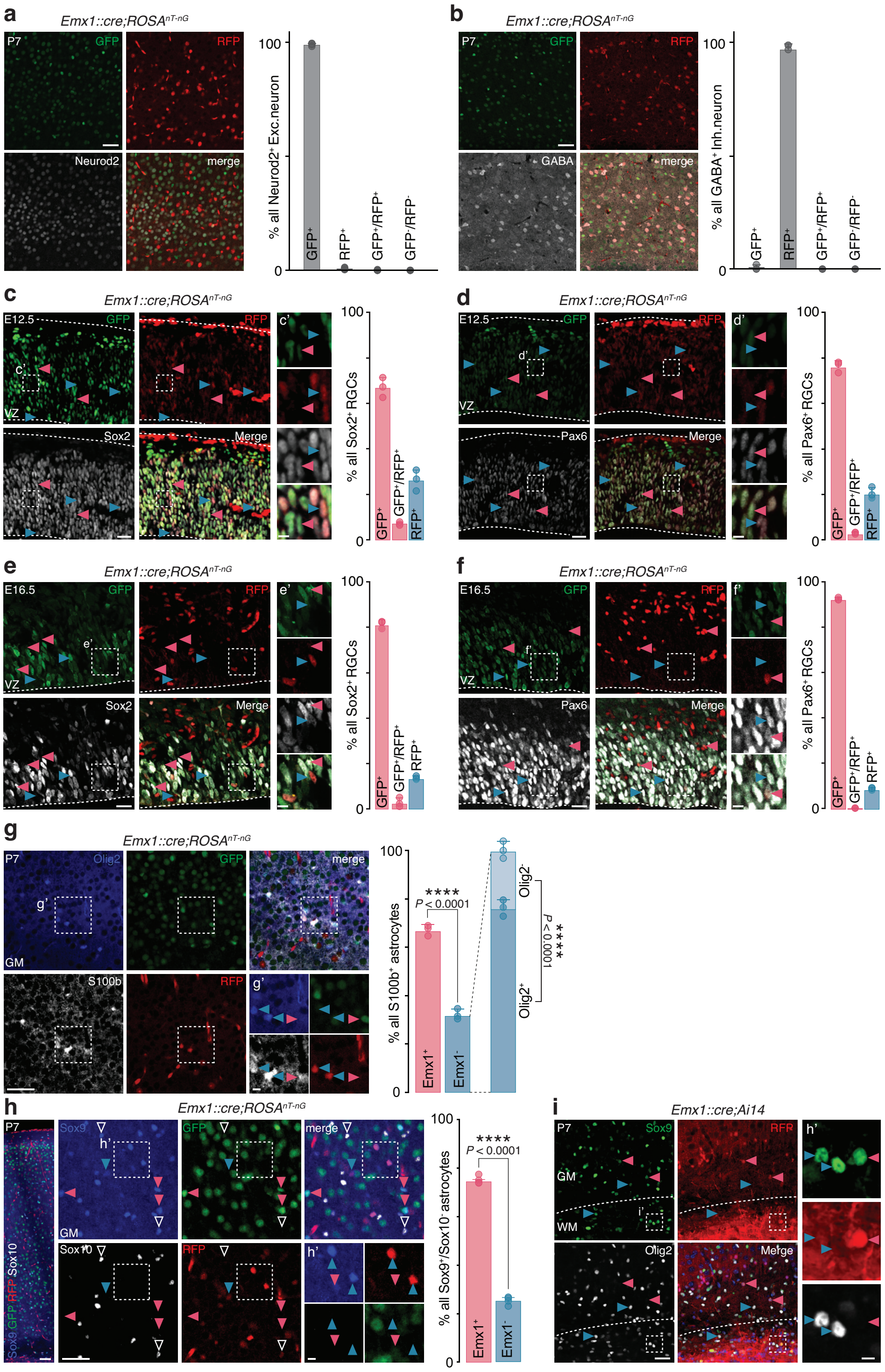

Supplementary Fig. 9 | Lineage tracing of *Emx1*⁺ RGC with reporter mouse lines. a-b, Left: P7 cortex from an *Emx1::cre;ROSA*$^{nT\text{-}nG}$ transgenic mouse, immunostained for Neurod2 (**a**) to label all excitatory neurons, or for GABA (**b**) to label all inhibitory neurons. Right: quantification of the proportion of GFP^{+} (*Emx1*$^{+}$), RFP^{+} (*Emx1*$^{-}$), GFP^{+}/RFP^{+} (*Emx1*$^{+}$), and GFP^{-}/RFP^{-} (unlabeled) excitatory neurons (**a**) or inhibitory neurons (**b**). n = 3 animals. **c-f**, Left: VZ and SVZ from *Emx1::cre;ROSA*$^{nT\text{-}nG}$ transgenic mice at E12.5 (**c**, **d**) or E16.5 (**e**, **f**), immunostained for Sox2 (**c**, **e**) or Pax6 (**d**, **f**) to label all RGCs. Right: quantification of the proportion of GFP^{+} (*Emx1*$^{+}$: pink arrowheads), RFP^{+} (*Emx1*$^{-}$: blue arrowheads), and GFP^{+}/RFP^{+} (*Emx1*$^{+}$) RGCs. n = 3 animals. **g**, Left: P7 cortex from *Emx1::cre;Rosa*$^{nT\text{-}nG}$ transgenic mouse ($S100b^{+}$/*Emx1*$^{+}$/$Olig2^{-}$: pink arrowheads; $S100b^{+}$/*Emx1*$^{-}$/$Olig2^{+}$: blue arrowheads). Right: Quantifications of $S100b^{+}$/*Emx1*$^{+}$ and $S100b^{+}$/*Emx1*$^{-}$ astrocytes in the entire cortical column. Olig2 expression is further quantified in all $S100b^{+}$/*Emx1*$^{-}$ astrocytes. n = 3 animals; two-tailed *t*-test. **h**, Left: P7 cortex from *Emx1::cre;Rosa*$^{nT\text{-}nG}$ transgenic mouse immunostained for Sox9 and Sox10. Right: examples of $Sox9^{+}/Sox10^{-}$ astrocytes ($Sox9^{+}/Sox10^{-}$/*Emx1*$^{+}$: pink arrowheads; $Sox9^{+}/Sox10^{-}$/*Emx1*$^{-}$: blue arrowheads. White empty arrowed indicate $Sox10^{+}$ oligodendrocytes. Quantifications of $Sox9^{+}/Sox10^{-}$/*Emx1*$^{+}$ and $Sox9^{+}/Sox10^{-}$/*Emx1*$^{-}$ astrocytes across the cortical column. n = 5 animals; two-tailed *t*-test. **i**, Examples of $Sox9^{+}$ astrocytes in P7 cortex of *Emx1::cre;Ai14* transgenic mice ($Sox9^{+}$/*Emx1*$^{+}$/Olig2: pink arrowheads; $Sox9^{+}$/*Emx1*$^{-}$/$Olig2^{+}$: blue arrowheads). Scale bars: 100 μm (column in h), 50 μm (overviews) and 10 μm (magnifications). ****$p<0.0001$. Values are shown as mean ± s.d.. VZ: ventricular zone; WM: white matter; GM: grey matter. Source data are provided as a Source Data file.

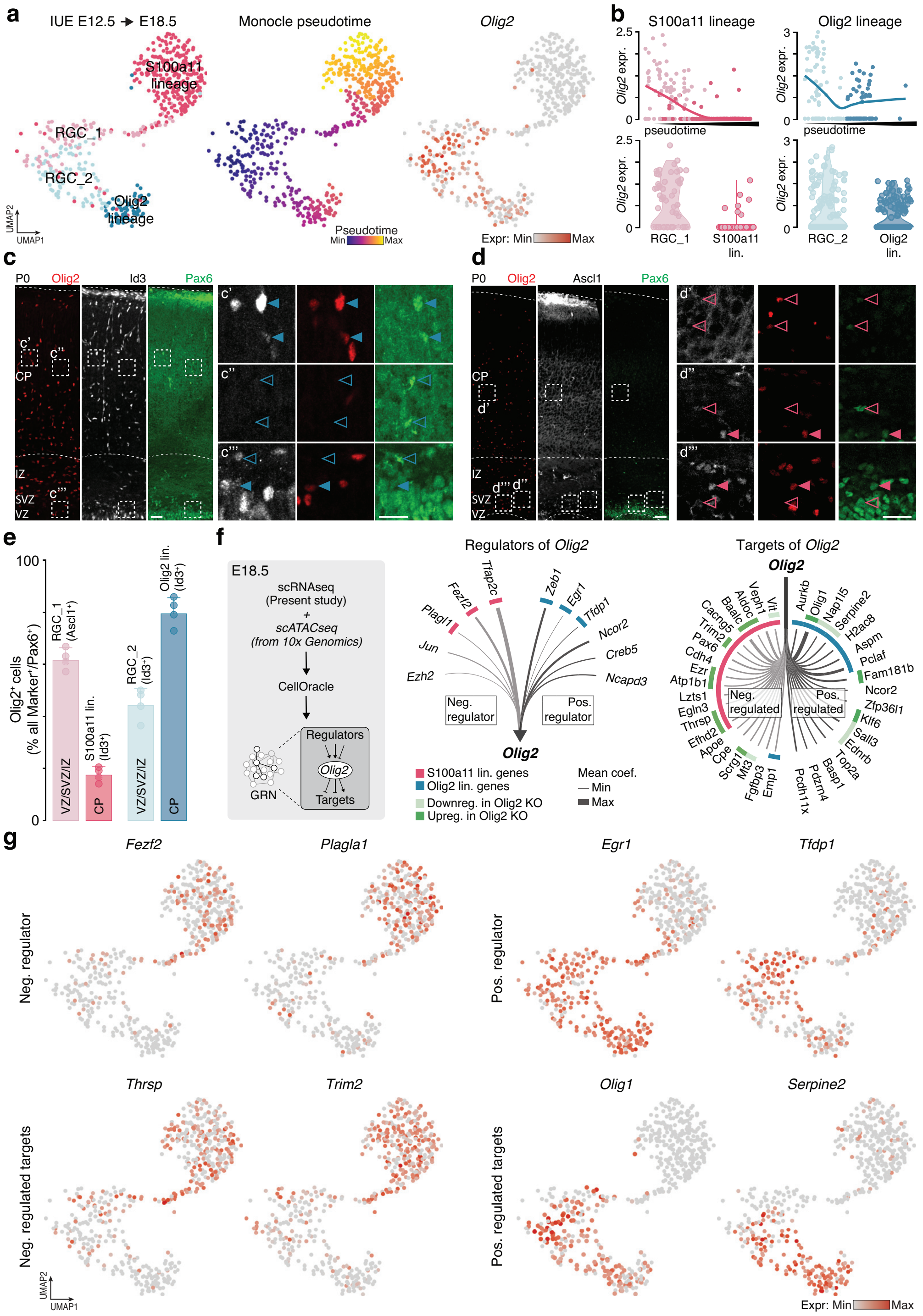

Supplementary Fig. 10 | Olig2 is expressed by both RGC subtypes but maintained only in the Olig2 lineage. a, UMAP representations of E18.5 RGCs and astrocytes (from Fig. 3b), color-coded by cell type (left), Monocle pseudotime (middle), and Olig2 expression (right). **b**, Olig2 expression along Monocle pseudotime in RGCs and astrocytes, showing its downregulation in the S100a11 lineage (left) and its maintenance in the Olig2 lineage (right). **c-d**, P0 cortical columns immunostained for Id3 (**c**) to label RGC_2 and Olig2 lineage astrocytes, and for Ascl1 (**d**) to label RGC_1, along with Olig2 and Pax6. Scale bars: 100 µm (overview) and 50 µm (magnification). **e**, Quantification of Olig2$^+$/Pax6$^+$ RGCs (in the VZ/SVZ/IZ) that are Ascl1$^+$ (RGC_1) or Id3$^+$ (RGC_2), as well as newly born astrocytes (in the CP) that are Id3$^+$ (Olig2 lineage) or Id3$^-$ (S100a11 lineage). n = 4 animals. **f**, Left: Schematic of the approach used to identify the *Olig2* gene regulatory network (GRN) in mouse at E18.5. Center: Chord diagram showing the predicted upstream regulators of *Olig2*, with the width of the connecting lines representing the scaled mean coefficient score from CellOracle GRN analysis. Right: Chord diagram showing the predicted downstream targets of *Olig2*, with the width of the lines representing the scaled mean coefficient score from CellOracle analysis. Negative (left) and positive (right) regulatory interactions are indicated, with transcription factors expressed by S100a11 and Olig2 lineage astrocytes shown in pink and blue, respectively. Genes that are downregulated and upregulated following *Olig2* knockout are highlighted in light and dark green, respectively. **g**, UMAP representations of E18.5 RGCs and astrocytes showing the expression of selected upstream regulators (top) and downstream targets (bottom) from the CellOracle GRN analysis. Values are shown as mean ± s.d.. VZ: ventricular zone; SVZ: subventricular zone; IZ: intermediate zone; CP: cortical plate. Source data are provided as a Source Data file.

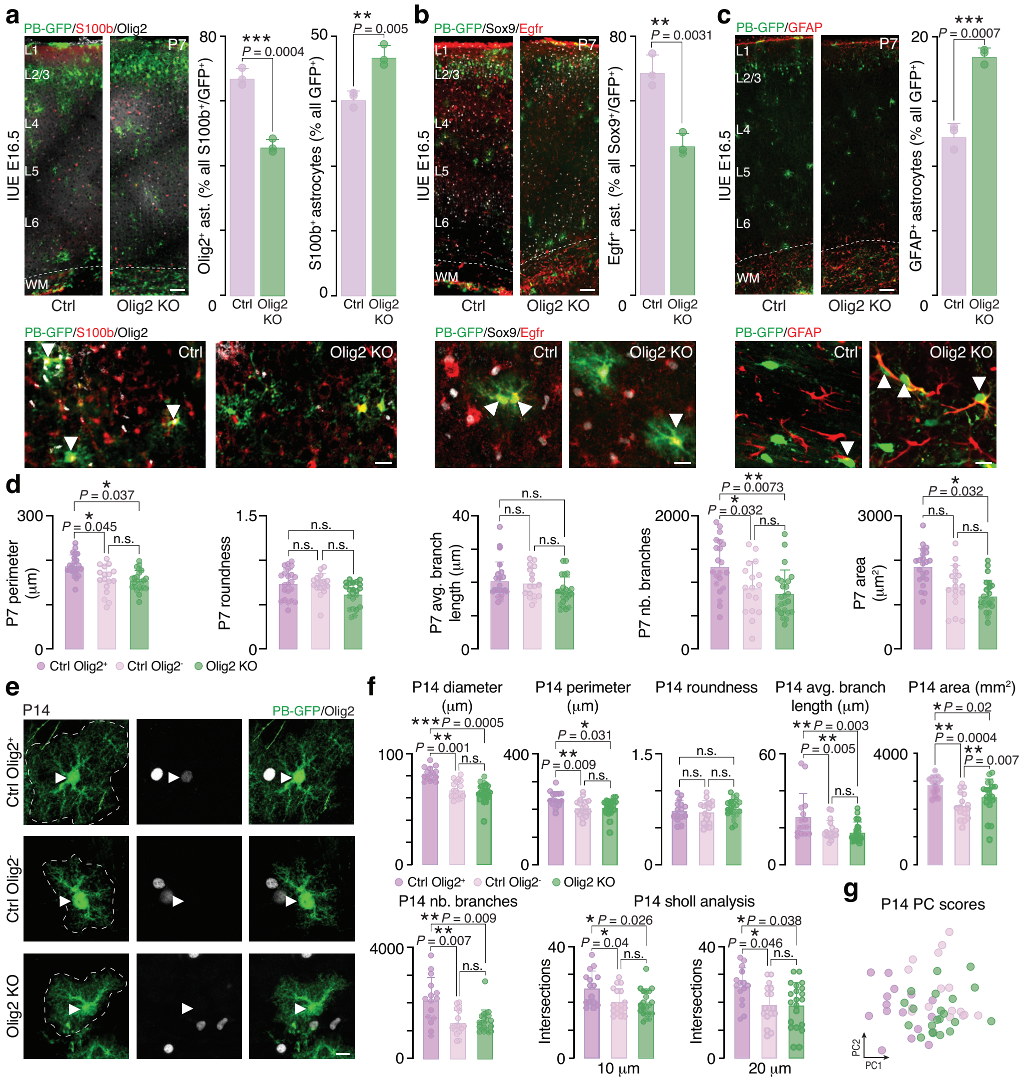

Supplementary Fig. 11 | Morphological alterations of astrocytes after *Olig2* knockout. a, Left: control (Ctrl) and *Olig2* knockout (Olig2 KO) cortical columns stained for S100b and Olig2. Bottom: magnified view showing colocalization (arrowhead) between the markers. Middle: fraction of Olig2$^+$ astrocytes in Ctrl and Olig2 KO cortices. Right: fraction of S100b$^+$ astrocytes among all GFP$^+$ electroporated cells. Scale bars: 100 µm (overview) and 10 µm (magnification). n = 3 animals; two-tailed *t*-test. **b**, Left: Ctrl and Olig2 KO cortical columns stained for Sox9 and Egfr. Bottom: magnified view showing colocalization (arrowhead) between the markers. Right: fraction of Egfr$^+$ astrocytes in Ctrl and Olig2 KO cortices. Scale bars: 100 µm (overview) and 10 µm (magnification). n = 3 animals; two-tailed *t*-test. **c**, Left: Ctrl and Olig2 KO cortical columns stained for GFAP. Bottom: magnified view showing colocalization (arrowhead) between the markers. Right: fraction of GFAP$^+$ astrocytes in Ctrl and Olig2 KO cortices. Scale bars: 100 µm (overview) and 10 µm (magnification). n = 3 animals; two-tailed *t*-test. **d**, Morphological parameters of P7 astrocytes used for PCA analysis (see Fig. 4h). n = 62 astrocytes, coming from 6 individual animals (3 for Ctrl and 3 for Olig2 KO); one-way ANOVA with post hoc Tukey test. **e**, P14 electroporated astrocytes in Ctrl and Olig2 KO cortices, labeled with Olig2 and classified into 3 groups: Ctrl Olig2$^+$, Ctrl Olig2$^-$, and Olig2 KO. Dashed lines indicate astrocyte territories. Scale bar: 10 µm. **f**, Morphological parameters of P14 astrocytes used for PCA analysis. n = 53 astrocytes, coming from 6 individual animals (3 for Ctrl and 3 for Olig2 KO); one-way ANOVA with post hoc Tukey test. **g**, PCA plot showing clustering of Ctrl Olig2$^+$, Ctrl Olig2$^-$, and Olig2 KO astrocytes by morphological parameters at P14. n.s. not significant, *p< 0.05, **p< 0.01, ***p<0.001. Values are shown as mean ± s.d.. WM: white matter; L: layer; IUE: *in utero* electroporation. Source data are provided as a Source Data file.

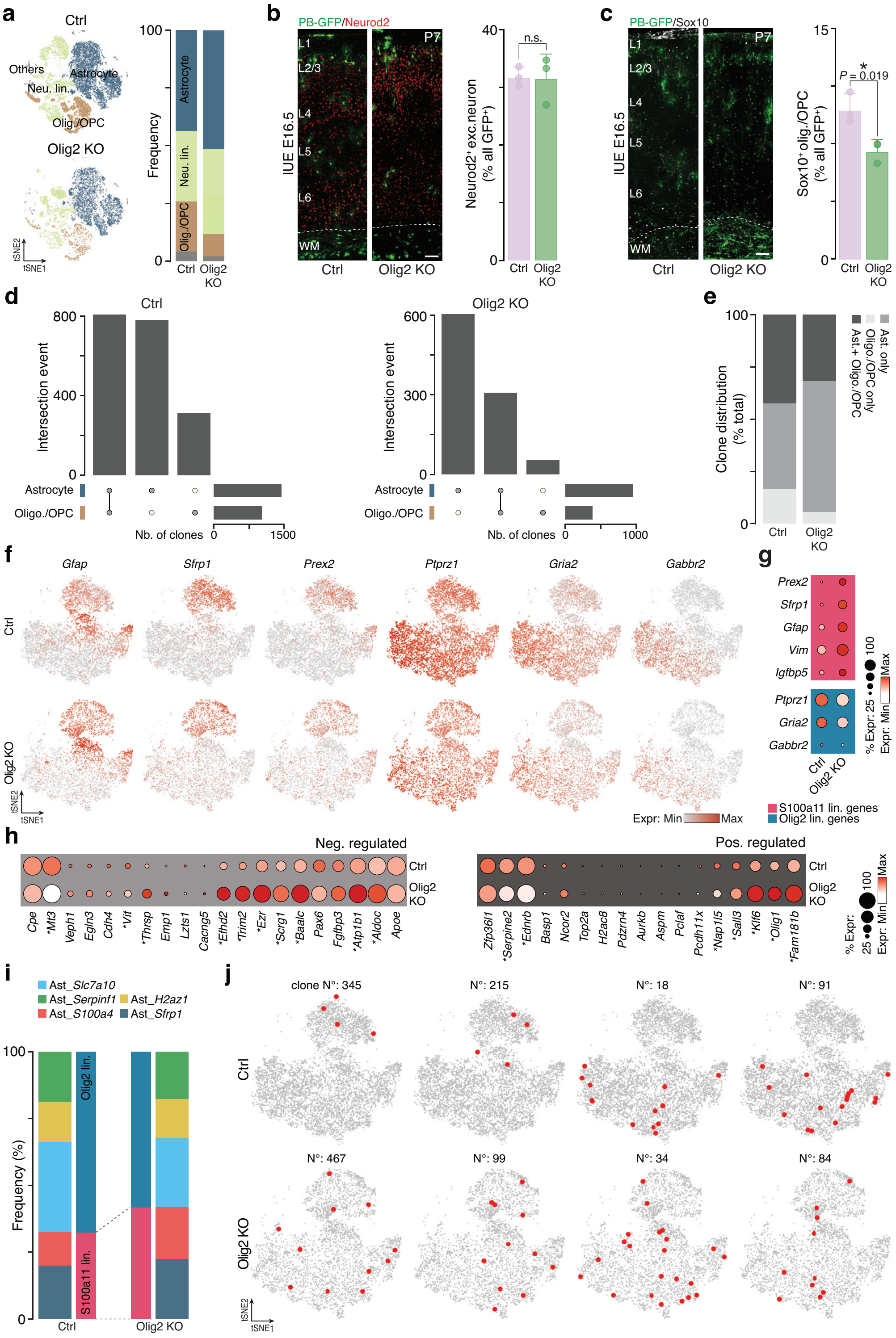

Supplementary Fig. 12 | Alterations in transcriptome, subtype composition, and clone distribution following *Olig2* knockout. a, Left: tSNE representations of cell types in control (Ctrl) and *Olig2* knockout (Olig2 KO) datasets. Right: proportion of each identified cell type. **b-c**, Left: Neurod2 (**b**) and Sox10 (**c**) staining in Ctrl and Olig2 KO cortical columns. Right: Fraction of Neurod2$^+$ excitatory neurons (**b**) and Sox10$^+$ oligodendrocytes/OPCs (**c**) among all GFP$^+$ electroporated cells. n = 3 animals; two-tailed t-test. Scale bars: 100 µm. **d**, UpSet plot showing the number of clones shared or unique between astrocytes and oligodendrocytes/OPCs in Ctrl (left) and Olig2 KO (right) conditions. Top bar graphs indicate observed intersections; right bar graphs show clone counts per cell subtype. **e**, Proportion of clones unique to astrocytes (Ast. only), oligodendrocytes/OPCs (Olig./OPC only), and mixed clones (Ast.+Oligo./OPC) in Ctrl and Olig2 KO conditions. **f-g**, tSNE plots (**f**) and dot plot (**g**) displaying the expression of representative genes upregulated in Ctrl and upregulated after Olig2 KO. **h**, Expression of *Olig2* regulators and targets in Ctrl and Olig2 KO conditions. * indicates genes differentially expressed between the two conditions. **i**, Relative proportion of each astrocyte subtype in Ctrl and Olig2 KO conditions. **j**, Examples of clones in Ctrl and Olig2 KO conditions. In Ctrl, clones are mainly restricted to either Olig2 lineage or S100a11 lineage astrocytes (top), while in Olig2 KO, clones are shared between the two lineages (bottom). n.s. not significant, *p<0.05. Values are shown as mean ± s.d.. WM: white matter; L: layer; IUE: *in utero* electroporation. Source data are provided as a Source Data file.

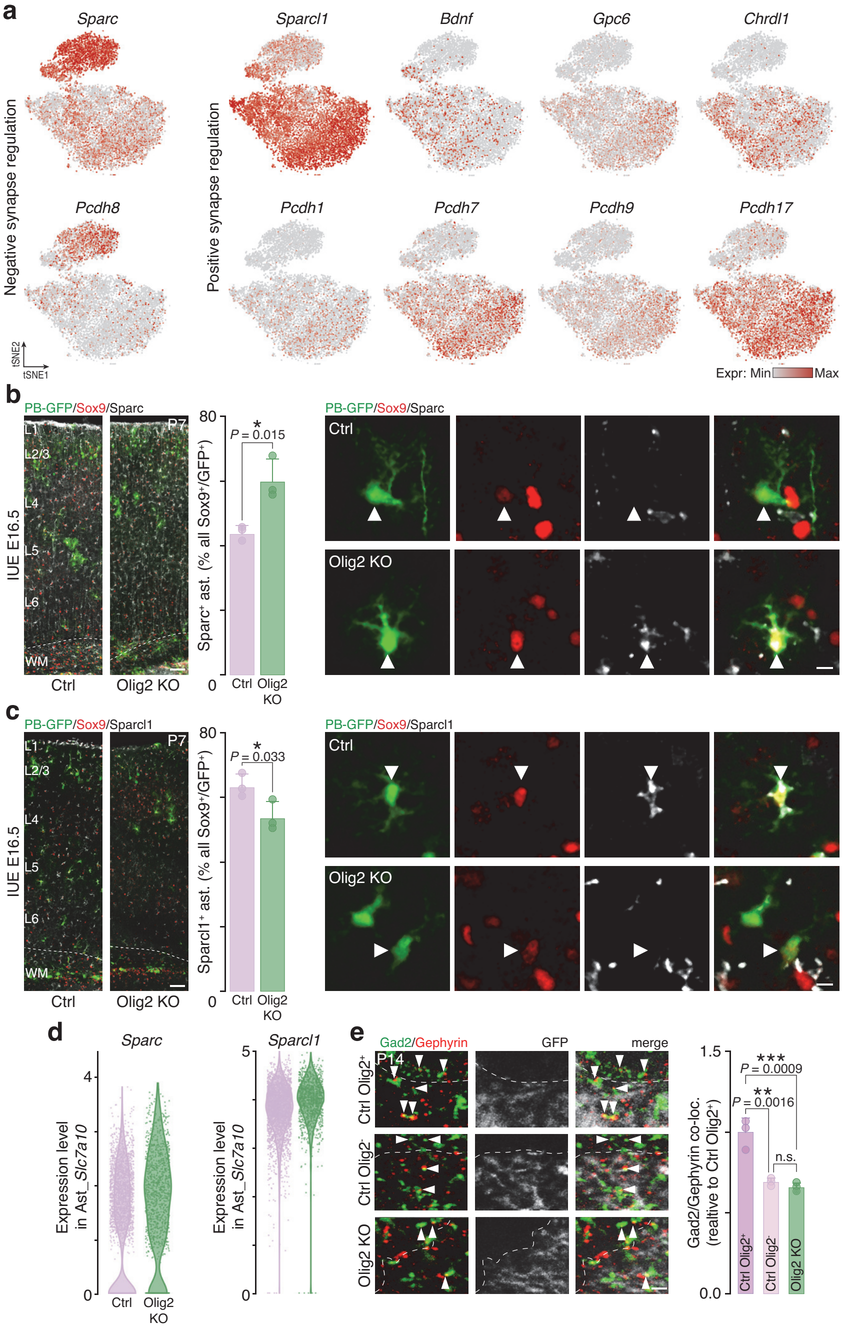

Supplementary Fig. 13 | S100a11 and Olig2 lineage astrocytes differentially express genes that regulate synapse formation. a, tSNE plots showing the expression of selected genes known to negatively (left) and positively (right) regulate synapse formation. Notably, S100a11 lineage astrocytes highly express genes associated with negative effects on synapse formation, whereas Olig2 lineage astrocytes primarily express genes that promote synaptogenesis. **b-c**, Left: Ctrl and Olig2 KO cortical columns stained for Sox9 and either Sparc (**b**) or Saprcl1 (**c**). Middle: fraction of Sparc^{+} or Sparcl1^{+} astrocytes in Ctrl and Olig2 KO cortices. Right: magnified view showing colocalization (arrowhead) between the markers. Scale bars: 100 µm (overview) and 10 µm (magnification). n = 3 animals; two-tailed t-test. **d**, Expression of *Sparc* and *Sparcl1* in the scRNA-seq dataset, comparing Ctrl and Olig2 KO conditions in the Ast_*Slc7a10* subtype. **e**, Left: inhibitory synapses within the GFP^{+} astrocyte territory for Ctrl Olig2^{+}, Ctrl Olig2^{-}, and Olig2 KO astrocytes at P14. Scale bar: 2 µm. Dashed lines indicate astrocyte boundaries, and arrowheads highlight co-localized synaptic puncta. Right: quantification of co-localized puncta in GFP^{+} astrocyte territories. n = 3 animals; one-way ANOVA with post hoc Tukey test. Scale bar: 2 µm. n.s. not significant, *p<0.05, **p<0.01, ***p<0.001. Values are shown as mean ± s.d. WM: white matter; L: layer; IUE: *in utero* electroporation. Source data are provided as a Source Data file.

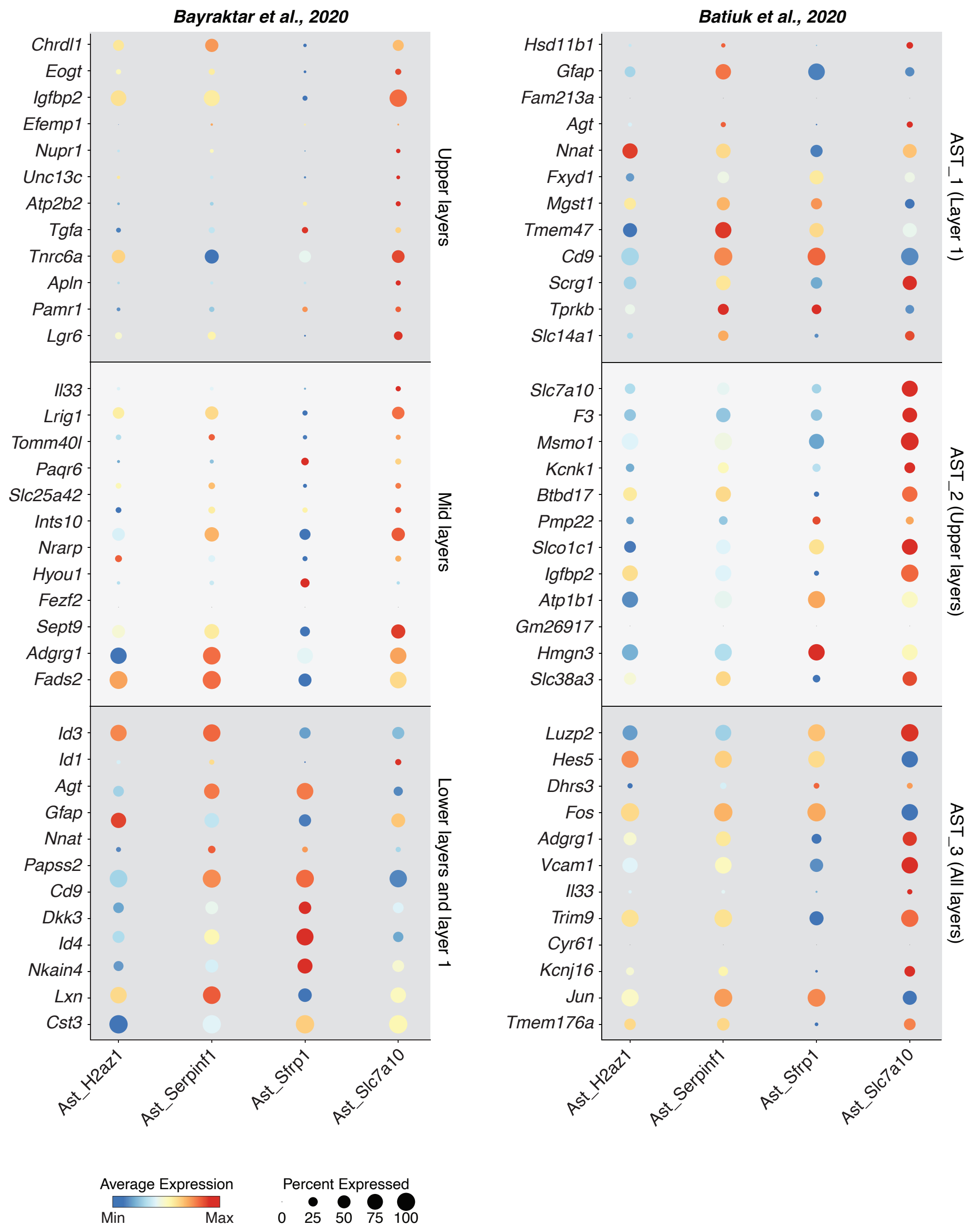

Supplementary Fig. 14 | Molecular comparison of the four grey matter astrocyte subtypes with previously reported studies. Dot plots showing the expression of 12 marker genes previously identified by *Bayraktar et al.* (left) and *Batiuk et al.* (right) as defining distinct astrocyte subtypes. Bayraktar et al. classified astrocytes into Upper layers, Mid layers, and Lower layers/Layer 1 subtypes, while *Batiuk et al.* categorized them as AST_1, AST_2, and AST_3. The dot plots depict how these previously identified markers are expressed across the grey matter astrocyte subtypes defined in this study (Ast_*H2az1*, Ast_*Serpinf1*, Ast_*Sfrp1* and Ast_*Slc7a10*), allowing for molecular comparisons between classification systems.
