# Supplementary material for: Dual lineage origins contribute to neocortical astrocyte diversity
Source: Nat Commun. 2025 Jul 30;16:6992. doi: 10.1038/s41467-025-61829-4 (PMC12310952; doi:10.1038/s41467-025-61829-4)
Supplement: Supplementary file 2 — Description of Additional Supplementary Files [file 41467_2025_61829_MOESM2_ESM.pdf]

Description of Additional Supplementary Files

Supplementary Data 1 | List of genes used for calculating cell type-specific and proliferation scores.

Supplementary Data 2 | Differential gene expression analysis between astrocytes subtypes. This data presents the differential gene expression analysis comparing astrocyte subtypes, as shown in Fig. 1b. Results are visualized in Fig. 1c. Statistical significance was assessed using a two-sided Wilcoxon Rank Sum test with Bonferroni correction for multiple testing. All p-values are reported in the table.

Supplementary Data 3 | List of genes included in the MERFISH panel used in this study.

Supplementary Data 4 | Gene cascade analysis along astrocyte trajectories. This data presents the genes possess differential dynamics along astrocyte trajectories (S100a11 and Olig2) as shown in Fig. 2c. The results are visualized in Fig. 2d. The table also includes genes commonly expressed during the early phase of development (shared trajectory).

Supplementary Data 5 | Differential gene expression analysis between RGC subtypes at E12.5 and E18.5. This data presents the differential gene expression analysis comparing RGC subtypes (RGC_1 and RGC_2), as shown in Fig. 3b. Results are visualized in Fig. 3j (E12.5) and Fig. 3e (E18.5). Statistical significance was assessed using a two-sided Wilcoxon Rank Sum test with Bonferroni correction for multiple testing. All p-values are reported in the table.

Supplementary Data 6 | CellOracle gene regulatory network (GRN) analysis. The GRN analysis was performed using all E18.5 RGCs, including both RGC_1 and RGC_2 clusters. In the table, the "source" column refers to the upstream regulator, while the "target" column lists genes predicted to be either positively (positive coef) or negatively (negative coef) regulated by the source gene. To assess the significance of inferred TF–target interactions, two-sided empirical p-values were calculated. No multiple testing correction was applied unless otherwise specified.

Supplementary Data 7 | Differential gene expression analysis between Ctrl and Olig2 KO astrocytes. This data presents the differential gene expression analysis comparing control and Olig2 knockout (KO) astrocytes, as shown in Fig. 4i. Results are visualized in Fig. 4j. Statistical significance was assessed using a two-sided Wilcoxon Rank Sum test with Bonferroni correction for multiple testing. All p-values are reported in the table.

Supplementary Data 8 | Gene sets for astrocyte functional scoring. This table contains gene sets associated with key astrocyte functions, including blood–brain barrier regulation, neuron projection development, brain homeostasis, astrocyte activation, synapse formation, and neuronal metabolic support.
