# Supplementary material for: Dual lineage origins contribute to neocortical astrocyte diversity
Source: Nat Commun. 2025 Jul 30;16:6992. doi: 10.1038/s41467-025-61829-4 (PMC12310952; doi:10.1038/s41467-025-61829-4)
Supplement: Supplementary file 11 — Reporting Summary [file 41467_2025_61829_MOESM11_ESM.pdf]

nature portfolio

Corresponding author(s): Bocchi Riccardo

Last updated by author(s): Jun 13, 2025

Reporting Summary

Nature Portfolio wishes to improve the reproducibility of the work that we publish. This form provides structure for consistency and transparency in reporting. For further information on Nature Portfolio policies, see our Editorial Policies and the Editorial Policy Checklist.

Statistics

For all statistical analyses, confirm that the following items are present in the figure legend, table legend, main text, or Methods section.

n/a	Confirmed	
☐	☒	The exact sample size (*n*) for each experimental group/condition, given as a discrete number and unit of measurement
☐	☒	A statement on whether measurements were taken from distinct samples or whether the same sample was measured repeatedly
☐	☒	The statistical test(s) used AND whether they are one- or two-sided<br>*Only common tests should be described solely by name; describe more complex techniques in the Methods section.*
☒	☐	A description of all covariates tested
☐	☒	A description of any assumptions or corrections, such as tests of normality and adjustment for multiple comparisons
☐	☒	A full description of the statistical parameters including central tendency (e.g. means) or other basic estimates (e.g. regression coefficient) AND variation (e.g. standard deviation) or associated estimates of uncertainty (e.g. confidence intervals)
☐	☒	For null hypothesis testing, the test statistic (e.g. *F*, *t*, *r*) with confidence intervals, effect sizes, degrees of freedom and *P* value noted<br>*Give P values as exact values whenever suitable.*
☒	☐	For Bayesian analysis, information on the choice of priors and Markov chain Monte Carlo settings
☒	☐	For hierarchical and complex designs, identification of the appropriate level for tests and full reporting of outcomes
☒	☐	Estimates of effect sizes (e.g. Cohen's *d*, Pearson's *r*), indicating how they were calculated

Our web collection on statistics for biologists contains articles on many of the points above.

Software and code

Policy information about availability of computer code

Data collection	BD FACSAria™ Fusion & BD FACSDiva Software, version v9.0; Zeiss Laser-scanning confocal LSM 800 Airyscan and Axio Imager A2 equipped with Zen software (v2.3); Illumina NovaSeq6000.
Data analysis	Confocal images were analyzed with ImageJ software (version 2.14.0/1.54f).<br>Statistical analysis was performed using GraphPad Prism (version 10.0.1).<br>Primary processing of raw scRNA-seq sequencing data to fastq files was carried out with Illumina's bcl2fastq (version 2.20.0.422) and alignment was performed using Cell Ranger (version 6.0.1). R(version 4.2.1) with Seurat (version 4.3.0.1) package was used to analyze scRNA-seq datasets. Harmony (version 0.1.1) package was used for datasets integration. Monocle (version 3) & URD (version 1.1.1) were used to calculate pseudotime and trajectories. Gene regulatory analysis was performed using CellOracle (version 0.10.15). Cell cycle analysis was performed using tricycle (version 1.4.0). Lineage barcode analysis was performed using scripts from https://github.com/mayer-lab/Bandler-et-al_lineage.

For manuscripts utilizing custom algorithms or software that are central to the research but not yet described in published literature, software must be made available to editors and reviewers. We strongly encourage code deposition in a community repository (e.g. GitHub). See the Nature Portfolio guidelines for submitting code & software for further information.

Data

Policy information about availability of data

All manuscripts must include a data availability statement. This statement should provide the following information, where applicable:

- Accession codes, unique identifiers, or web links for publicly available datasets
- A description of any restrictions on data availability
- For clinical datasets or third party data, please ensure that the statement adheres to our policy

The scRNA-seq data generated in this study have been deposited in the Sequence Read Archive under accession code PRJNA1027603. The MERFISH data generated in this study have been deposited in Zenodo under accession code 15632740. The processed datasets can be browsed at http://www.bocchilab.ch/Zhou_et_al_2025. The scRNA-seq datasets from Di Bella et al., were obtained from Gene Expression Omnibus under accession code GSE153164. The human scRNA-seq datasets from Trevino et al., were obtained from Gene Expression Omnibus under accession code GSE162170. The reptile scRNA-seq datasets from Tosches et al., were obtained from Sequence Read Archive under accession code PRJNA408230. The bird scRNA-seq datasets from Zaremba et al., were obtained from heiDATA. The Visium and scRNA-seq datasets generated from Bocchi et al., were obtained from Sequence Read Archive under accession PRJNA1125165. The mouse scRNA-seq datasets from Endo et al., were obtained from Gene Expression Omnibus under accession code GSE198027. Source data are provided with this paper.

Research involving human participants, their data, or biological material

Policy information about studies with human participants or human data. See also policy information about sex, gender (identity/presentation), and sexual orientation and race, ethnicity and racism.

Reporting on sex and gender	NA
Reporting on race, ethnicity, or other socially relevant groupings	NA
Population characteristics	NA
Recruitment	NA
Ethics oversight	NA

Note that full information on the approval of the study protocol must also be provided in the manuscript.

Field-specific reporting

Please select the one below that is the best fit for your research. If you are not sure, read the appropriate sections before making your selection.

[x] Life sciences [ ] Behavioural & social sciences [ ] Ecological, evolutionary & environmental sciences

For a reference copy of the document with all sections, see nature.com/documents/nr-reporting-summary-flat.pdf

Life sciences study design

All studies must disclose on these points even when the disclosure is negative.

Sample size	No statistical methods were used to predetermine sample size. After removing low-quality cells, 111,267 cells were retained for the integrated dataset; 8,958 cells were kept for the E18.5 electroporation dataset; 1,001 cells were kept for the E14 MERFISH dataset; 32,122 cells were retained for the P7 MERFISH dataset; 103,421 cells were retained for the adult MERFISH dataset.
Data exclusions	No data were excluded.
Replication	E12.5-P7 dataset: 1 sample from 1 brain; E16.5-P7 control dataset: 2 samples from 1 brain;<br>E16.5-P7 Olig2 knockout dataset: 1 sample from 1 brain; E12.5-E18.5 dataset: 1 sample from 1 brain.<br>E14 MERFISH dataset: 1 section from 1 brain.<br>P7 MERFISH dataset: 1 section from 1 brain.<br>adult MERFISH dataset: 1 section from 1 brain.
Randomization	Mice were chosen in a random manner and distributed among various experimental groups. There was no further randomization during the process of data collection.
Blinding	When applicable, the data collection and analysis process were conducted without the investigators knowing the group assignments (blinded). After the analysis was finished, the group assignments were disclosed.

Reporting for specific materials, systems and methods

We require information from authors about some types of materials, experimental systems and methods used in many studies. Here, indicate whether each material, system or method listed is relevant to your study. If you are not sure if a list item applies to your research, read the appropriate section before selecting a response.

Materials & experimental systems

n/a	Involved in the study
☐	☒ Antibodies
☒	☐ Eukaryotic cell lines
☒	☐ Palaeontology and archaeology
☐	☒ Animals and other organisms
☒	☐ Clinical data
☒	☐ Dual use research of concern
☒	☐ Plants

Methods

n/a	Involved in the study
☒	☐ ChIP-seq
☐	☒ Flow cytometry
☒	☐ MRI-based neuroimaging

Antibodies

Antibodies used

The primary antibodies used, along with their dilutions, were as follows: chicken anti-GFP (Invitrogen, A10262, 1:1000), rat anti-RFP (Chromotek, 5F8, 1:500), rabbit anti-Sox9 (Merck Millipore, AB5535, 1:500), rabbit anti-Neurod2 (Abcam, ab104430, 1:500), rabbit anti-Pax6 (Covance, PRB-278P , 1:500), rabbit anti-GABA (Sigma-Aldrich, A2052, 1:500), rabbit anti-S100b (Abcam, ab41548, 1:500), goat anti-Olig2 (R&D Systems, AF2418, 1:200), rabbit anti-Sox2 (Abcam, ab97959, 1:500), guineapig anti-VGLUT1 (Synaptic System, 135304, 1:1000), mouse anti-PSD95 (Millipore, MAB1596, 1:600), goat anti-EGFR (R&D Systems, FAB9577B, 1:1000), goat anti-Sparc (R&D Systems, AF942; 1:200), goat anti-sparcl1(R&D Systems, AF2836, 1:1000), mouse anti-Gad2 (Merck, MAB5406; 1:500), guineapig anti-Gephyrin (Synaptic Systems, 147318; 1:200), goat anti-Sox10 (Santa Cruz, sc-17342, 1:500), rat anti-Sox2 (Thermo Fisher, 14-9811-82, 1:500), rabbit anti-Id3 (Cell Signaling Technology, #9837, 1:500), mouse anti-Ascl1 (BD Biosciences, 556604, 1:200), rat anti-Ki67 APC-conjugated (Thermo Fisher, 17-5698-82, 1:500), mouse anti-Pax6 (BD Bioscience, 561462, 1:500), rabbit anti-GFAP(Abcam, ab7260, 1:500).

Secondary antibodies are listed as follows (dilution 1:1000): Donkey anti-Rabbit IgG, Alexa Fluor 488 Invitrogen A11055; Donkey anti-Rabbit IgG, Alexa Fluor 647 Invitrogen A31573; Donkey anti Goat IgG, Alexa Fluor 488 Invitrogen A21206; Donkey anti-Rabbit IgG, Alexa Fluor 405 Invitrogen A48258; Donkey anti-Chicken IgG, Alexa Fluor 488 Invitrogen A78948; Donkey anti-Rat IgG, Alexa Fluor 594 Invitrogen A21209; Donkey anti-Goat IgG, Alexa Fluor 405 Abcam ab175664.

Validation

Following antibodies have been validated previously in mouse brain tissue:

- rabbit anti-Sox9 (Merck Millipore, AB5535, validated in PMID: 21256198),
- rabbit anti-Sox2 (Abcam, ab97959, validated in PMID: 38135757),
- goat anti-Olig2 (R&D Systems, AF2418, validated in PMID: 33606177),
- chicken anti-GFP (Invitrogen, A10262, validated in PMID: 35523590),
- rat anti-RFP (Chromotek, 5F8, validated in PMID: 36922596),
- rabbit anti-Neurod2 (Abcam, ab104430, validated in PMID: 37807845),
- rabbit anti-Pax6 (Covance, PRB-278P, validated in PMID: 36095192),
- rabbit anti-GABA (Sigma-Aldrich, A2052, validated in PMID: 12807990),
- rabbit anti-S100b (Abcam, ab41548, validated in PMID: 37837557),
- guineapig anti-VGLUT1 (Synaptic System, 135304, validated in PMID: 37985778),
- mouse anti-PSD95 (Millipore, MAB1596, validated in PMID: 25253857),
- goat anti-EGFR (R&D Systems, FAB9577B, validated in PMID: 38713624),
- goat anti-Sparc (R&D Systems, AF942, validated in PMID: 35253855),
- goat anti-sparcl1(R&D Systems, AF2836, validated in PMID: 35253855),
- mouse anti-Gad2 (Merck, MAB5406, validated in PMID: 25918648),
- guineapig anti-Gephyrin (Synaptic Systems, 147318, validated in PMID: 34580165),
- goat anti-Sox10 (Santa Cruz, sc-17342, validated in PMID: 28400715),
- rat anti-Sox2 (Thermo Fisher, 14-9811-82, validated in PMID: 39261744),
- rabbit anti-Id3 (Cell Signaling Technology, #9837, validated in PMID: 35614216),
- mouse anti-Ascl1 (BD Biosciences, 556604, validated in PMID: 30643018),
- rat anti-Ki67 APC-conjugated (Thermo Fisher, 17-5698-82, validated in PMID: 35045305).

Animals and other research organisms

Policy information about studies involving animals; ARRIVE guidelines recommended for reporting animal research, and Sex and Gender in Research

Laboratory animals	Female mice of the CD1 and C57BL/6 strains, obtained from Charles River Laboratory, were utilized in this study. The embryos were staged based on days post-coitus, with E0.5 defined as 12:00 on the day following the detection of a vaginal plug after overnight mating. For lineage tracing of Emx1+ RGCs, Emx1-IRES-Cre mice (B6.129S2-Emx1tm1(cre)Krj/J, Jackson number: 005628) were crossed with either Ai14 mice (B6.Cg-Gt(ROSA)26Sortm14(CAG-tdTomato)Hze/J, Jackson number: 007914) or ROSAnT-nG mice (B6;129S6-Gt(ROSA)26Sortm1(CAG-tdTomato*,-EGFP*)Ees/J, Jackson number: 023035). Brain tissue from these lines were kindly provided by Magdalena Götz laboratory. All mice were housed in the institutional animal facility, with a standard 12-hour light and 12-hour dark cycle and had access to food and water ad libitum. Both sexes were included in all the animal experiments unless stated otherwise.
Wild animals	no wild animals were used in this study.
Reporting on sex	This study did not take the gender into consideration, and embryos and postnatal pups were randomly chosen.
Field-collected samples	This study did not involve samples collected from the field.
Ethics oversight	All experimental procedures described in this study were conducted in full compliance with Swiss legislation and were approved by the Geneva Cantonal Veterinary Authority (authorization no. 34938) and the Service de la Consommation et des Affaires Vétérinaires of the Canton of Vaud (authorization no. VD3755).

Note that full information on the approval of the study protocol must also be provided in the manuscript.

Flow Cytometry

Plots

Confirm that:

- [x] The axis labels state the marker and fluorochrome used (e.g. CD4-FITC).
- [x] The axis scales are clearly visible. Include numbers along axes only for bottom left plot of group (a 'group' is an analysis of identical markers).
- [x] All plots are contour plots with outliers or pseudocolor plots.
- [ ] A numerical value for number of cells or percentage (with statistics) is provided.

Methodology

Sample preparation	Brains collected from E18.5 embryos were dissected on ice with Leibowitz medium with 5% FBS whereas brains collected from P7 pups were exposed and kept in bubbled EBSS with 5% FBS on ice then transferred to Hibernate A medium with 10% FBS and B27 (1:50 dilution), while being observed under a dissecting scope to identify the positive regions. GFP+ cortices were then dissociated using the Papain dissociation system following the recommended protocol from Worthington (#LK003150), and further processed with the gentleMACS Dissociator following the manufacturer's instructions. For more details, please see "Sample collection for scRNA-seq" section of the methods section.
Instrument	BD FACSAria™ Fusion
Software	BD FACSDiva Software, version v9.0
Cell population abundance	Cell population abundance was less than 1% of the total sorted cells.
Gating strategy	Initially, cell suspensions were gated based on forward scatter, then within this population, dead cells were excluded based on Dapi, and finally cells expressing GFP were collected.

- [ ] Tick this box to confirm that a figure exemplifying the gating strategy is provided in the Supplementary Information.
